# Tandem CAR-T cell therapy: recent advances and current challenges

**DOI:** 10.3389/fimmu.2025.1546172

**Published:** 2025-02-28

**Authors:** Sara Gómez-Melero, Fakhri Hassouneh, Isabel M. Vallejo-Bermúdez, Eduardo Agüera-Morales, Rafael Solana, Javier Caballero-Villarraso

**Affiliations:** ^1^ Maimonides Biomedical Research Institute of Cordoba (IMIBIC), Reina Sofia University Hospital, University of Cordoba, Cordoba, Spain; ^2^ Department of Biochemistry and Molecular Biology, Universidad of Cordoba, Cordoba, Spain; ^3^ Neurology Department, Reina Sofia University Hospital, Cordoba, Spain; ^4^ Clinical Analyses Department, Reina Sofia University Hospital, Cordoba, Spain

**Keywords:** chimeric antigen receptor, immunotherapy, cancer, antigen escape, dual receptor, multitarget

## Abstract

CAR-T cell therapy has revolutionized cancer treatment. However, despite the achievements of this approach, there are still clinical challenges to address, such as antigen loss and the design of an optimal CAR structure. Multi-targeted CAR-T therapies, including tandem CAR-T cells, have emerged as a strategy to overcome some of these limitations and improve outcomes. Tandem CAR-T cells are currently being evaluated in preclinical and clinical studies for the treatment of hematological malignancies and solid tumors, showing promising results. These CARs have demonstrated efficacy, safety, and a relatively low relapse rate in these studies. Research suggests that TanCAR-T cells can enhance the outcomes and benefits of CAR-T cell therapy. However, challenges such as identifying the ideal CAR construct, selecting appropriate targets, and improving transduction efficiency remain unresolved, and further research is essential to address these limitations. This review highlights the potential of tandem CAR-T cells as a cancer treatment, summarizing preclinical and clinical studies with this innovative therapy and emphasizing the importance of continued research to overcome its limitations and improve its effectiveness.

## 1 Introduction

Cancer is a complex group of disorders characterized by uncontrolled cell growth and the potential for metastasis, affecting millions of people worldwide each year. Addressing the abnormal cell growth in cancer requires tailored approaches such as chemotherapy, surgery, and radiation therapy (1). However, these conventional treatments, which aim to eliminate cancer cells, have significant limitations, and many patients with metastatic or recurrent disease continue to experience poor outcomes (2). Consequently, alternative long-term approaches are needed to effectively treat cancer, such as immunotherapy, which enhances the patient’s immune system by modulating immune responses and enabling the detection and destruction of the cancer cells (1).

Chimeric antigen receptor (CAR)-T cell immunotherapy has emerged as a promising treatment for cancer. CAR-T cell therapy involves the genetic modification of T cells obtained from the patient’ s blood to express synthetic receptors, followed by their infusion back into the patient (3). These synthetic constructs are designed to bind to specific antigens expressed on the surface of cancer cells, triggering the activation of the CAR-T cells and the subsequent killing of tumor cells. By integrating synthetic domains through viral vectors, CAR-T cells express these domains on their surface, enabling them to attack the cancer cells more precisely and address problems associated with older immunotherapies (1) (4) (5),,. As a result, CAR-T cell therapy holds significant promise for improving upon traditional cancer treatments, such as chemotherapy and radiation, while reducing associated toxicities (3).

Among immunotherapeutic approaches, CAR-T cell therapy has emerged as an extremely powerful tool, particularly in treating relapsed/refractory (r/r) B cell malignancies and multiple myeloma (MM). This success has led to the approval of six CAR-T cell products by the Food and Drug Administration (FDA) and the European Medicines Agency (EMA) (6, 7), including Yescarta, Tecartus, Kymriah, Breyanzi, Carvykti, and Abecma. These therapies are indicated for acute lymphoblastic leukemia (ALL), large B-cell lymphoma, follicular lymphoma, mantle cell lymphoma (MCL), marginal zone lymphoma, and MM (8). Moreover, in November 2024, Autolus Therapeutics plc announces that the FDA has granted marketing approval for AUCATZYL^®^ (obecabtagene autoleucel) for the treatment of adult patients with r/r B-cell precursor acute lymphoblastic leukemia (B-ALL) and marketing authorization applications are under review by the regulators in both the EU and the UK (9).

However, although CAR-T cell therapy has achieved groundbreaking outcomes in the treatment of hematological cancers, several challenges and limitations remain (1). The biological characteristics of solid tumors are more complex, posing significant obstacles that prevent CAR-T cells from exerting effective anti-tumor responses (10). Key hurdles in CAR-T cell therapy include antigen escape variants, off-tumor destruction of healthy tissues expressing tumor associated antigens (TAAs), limited CAR-T cell persistence, and functional exhaustion. These factors limit the ability of CAR-T cells to induce long-lasting remissions with a tolerable adverse effect profile (6, 11). Traditional CARs are unable to fully overcome these challenges, highlighting the need to improve CAR-T cells efficacy by targeting specific tumor surface antigens. Consequently, specialized CARs constructs with unique structures or improvements based on traditional designs are actively being researched (10).

One of the key challenges in CAR-T cell therapy is tumor resistance due to the limitations of single-antigen targeting CAR designs. To overcome this issue, multiple strategies have been developed to target multiple antigens in CAR-T cell treatments for both, hematological cancers and solid tumors (1). One approach to targeting multiple tumor antigens simultaneously is through the use of tandem CARs (TanCARs), which incorporate two single-chain variable fragments (scFvs) within a single CAR construct, sharing one intracellular signaling domain. This design increase the likelihood of durable remission (1, 12). Multiple studies indicate that dual-antigen targeting may reduce the risk of tumor antigen escape and enhance tumor cell-killing activity (13, 14). TanCARs aim to enhance the antitumor activity of CAR-T cells by increasing antigen coverage and can be repurposed for the simultaneous targeting of malignant cells and elements of the tumor microenvironment (6, 15). These advantages are particularly important in the context of solid tumors, where antigen expression heterogeneity often leads to drug resistance and immune escape when treated with CAR-T cells (16). Due to the lack of tumor-specific targets and the presence of physiological barriers, it remains challenging for patients with solid tumors to benefit from CAR-T therapy (17). Despite the fact that the clinical efficacy of CAR-T cells against solid tumors has been less promising, it has been demonstrated that T cells expressing TanCARs exhibit functional superiority, even in solid tumors such as glioblastoma (17, 18).

This review comprehensively explores the current state of multi-target CAR-T therapies, focusing on tandem CAR-T (TanCAR-T) therapies, and the latest advancements in clinical and preclinical studies across different cancers. We summarize recent innovations in CAR-T cell engineering aimed at improving clinical effectiveness, in both hematological malignancies and solid tumors, as well as the potential use of TanCAR-T cell therapies to overcome current limitations, including antigen escape.

## 2 Multi-target CAR-T cells

### 2.1 CAR-T cells structure

**Figure 1:**
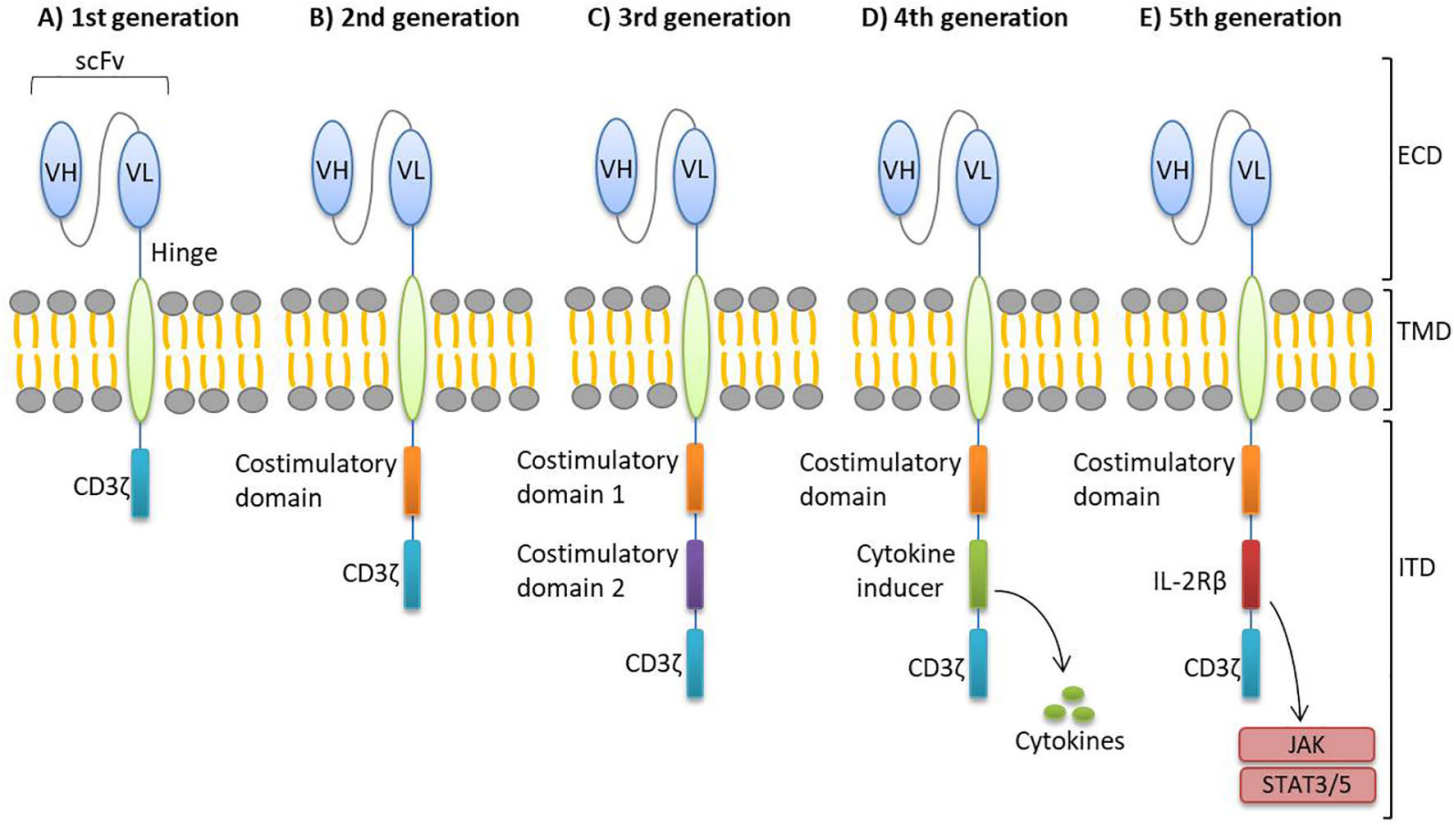
Generations of CAR-T cells. There are five generations of CARs, distinguished by their intracellular domains. **(A)** The first generation contains only the CD3ζ intracellular activation domain. **(B)** The second generation includes both the CD3ζ domain and a co-stimulatory domain. **(C)** Third generation CAR-T cells incorporate two co-stimulatory domains to enhance activation. **(D)** Fourth generation CARs feature a cytokine-inducing domain to improve immune response. **(E)** Fifth generation includes a truncated cytoplasmic IL-2Rβ domain, offering improved safety and controllability. CAR, chimeric antigen receptor; ECD, extracellular domain; ICD, intracellular domain; IL-2Rβ, IL-2 receptor β-chain; scFv, single-chain variable fragment; TMD, transmembrane domain; VH, variable heavy chain; VL, variable light chain.

The CAR structure is primarily composed of three functional domains: the extracellular domain, the transmembrane domain, and the intracellular signaling domain (19, 20). The intracellular signaling domain, essential for T cell activation, is typically derived from molecules such as 4-1BB, CD3ζ, and CD28. The extracellular antigen recognition domain consists of a scFv, which is derived from a monoclonal antibody or a phage display library. This scFv is functionally linked to the intracellular domain through a hinge or spacer domain and a transmembrane domain. The hinge or spacer domain enhances the flexibility of the scFv, facilitating better antigen binding (1, 3). Since the creation of CAR-T cell therapy in the late 1980s, five generations of CARs have been developed (**Figure 1**), each incorporating successive improvements in the intracellular signaling domain to improve T cell activation and therapeutic efficacy.

In first-generation CARs, the intracellular structure contains only one signaling domain, composed of the CD3ζ chain, which initiates T cell receptor signaling. Second-generation CARs introduced a co-stimulatory molecule, such as CD28 or 4-1BB (CD137), to improve the persistence and efficacy of CAR-T cells (7, 21). Third-generation CARs feature an intracellular domain containing two co-stimulatory molecules, commonly CD28 or 4-1BB as the first, and CD28, 4-1BB, or OXO40 (CD134) as the second (19, 22). This design aims to amplify T cell effector functions and cytotoxic activity upon antigen recognition. Fourth-generation CARs, also known as TRUCKs (T cells Redirected for Universal Cytokine-mediated Killing), incorporate a cytokine-inducing domain that promotes cytokine production after antigen recognition, helping to modulate immune responses (6, 10, 19, 23). Fifth-generation CARs, built on the second-generation design, include a truncated cytoplasmic IL-2 receptor β-chain (IL-2Rβ) domain, which provides a binding site for the transcription factor STAT3 and activates the JAK-STAT signaling pathway. Upon antigen recognition, these CARs trigger three synergistic signals through the T cell receptor (TCR) CD3ζ domain, the co-stimulatory domain, and the cytokine inducing JAK–STAT3/5 signaling (23, 24). Despite these advancements, none of the traditional CARs from the first to the fifth-generation can fully overcome the challenges associated with CAR-T cell therapy.

### 2.2 Multi-target approaches

**Figure 2:**
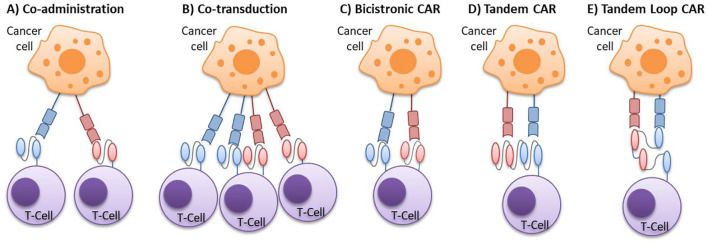
Multi-targeted CAR-T cell approaches. **(A)** Co-administration: a strategy involving a cocktail or sequential infusion of two single CAR-T cell products, each transduced independently with a different vector. **(B)** Co-transduction: a strategy generating a mixed population composed of two single CAR-T cells and one dual CAR-T cell, generated by the co-transduction with two vectors, each encoding a different CAR. **(C)** Bicistronic CAR: dual CAR-T cells produced by the transduction of a bicistronic vector, which introduces two separate CARs into a single cell. **(D)** Tandem CAR: bispecific CAR-T cells expressing a single CAR with two antigen-binding domains, where the VL-VH sequences of one scFv are directly linked to the VL-VH sequences of the other scFv. **(E)** Tandem Loop CAR: a variant of the tandem CAR, in which the VL-VH sequences of one scFv are intercalated with those of the other scFv, forming a bivalent loop CAR. CAR, chimeric antigen receptor; scFv, single-chain variable fragments; VH, variable heavy chain; VL, variable light chain.

There are several design options for CAR therapies to target multiple antigens concurrently (**Figure 2**). Multi-target CAR-T therapy can be achieved using either two pooled single CAR-T cell products with different antigen-binding specificities (through co-administration or co-transduction) or a single CAR-T cell product capable of targeting two different antigens (using bicistronic o tandem CAR-T designs) (25).

For two pooled single CAR-T cell products approaches, one strategy is the co-administration of two or more cell populations, each expressing different single-target CARs. These populations can be infused either together or sequentially. Another approach is the co-transduction method, where T cells are simultaneously transduced with two different CAR constructs, resulting in three CAR-T cell subsets: dual CAR-expressing cells and two types of single CAR-expressing cells.

**Figure 3:**
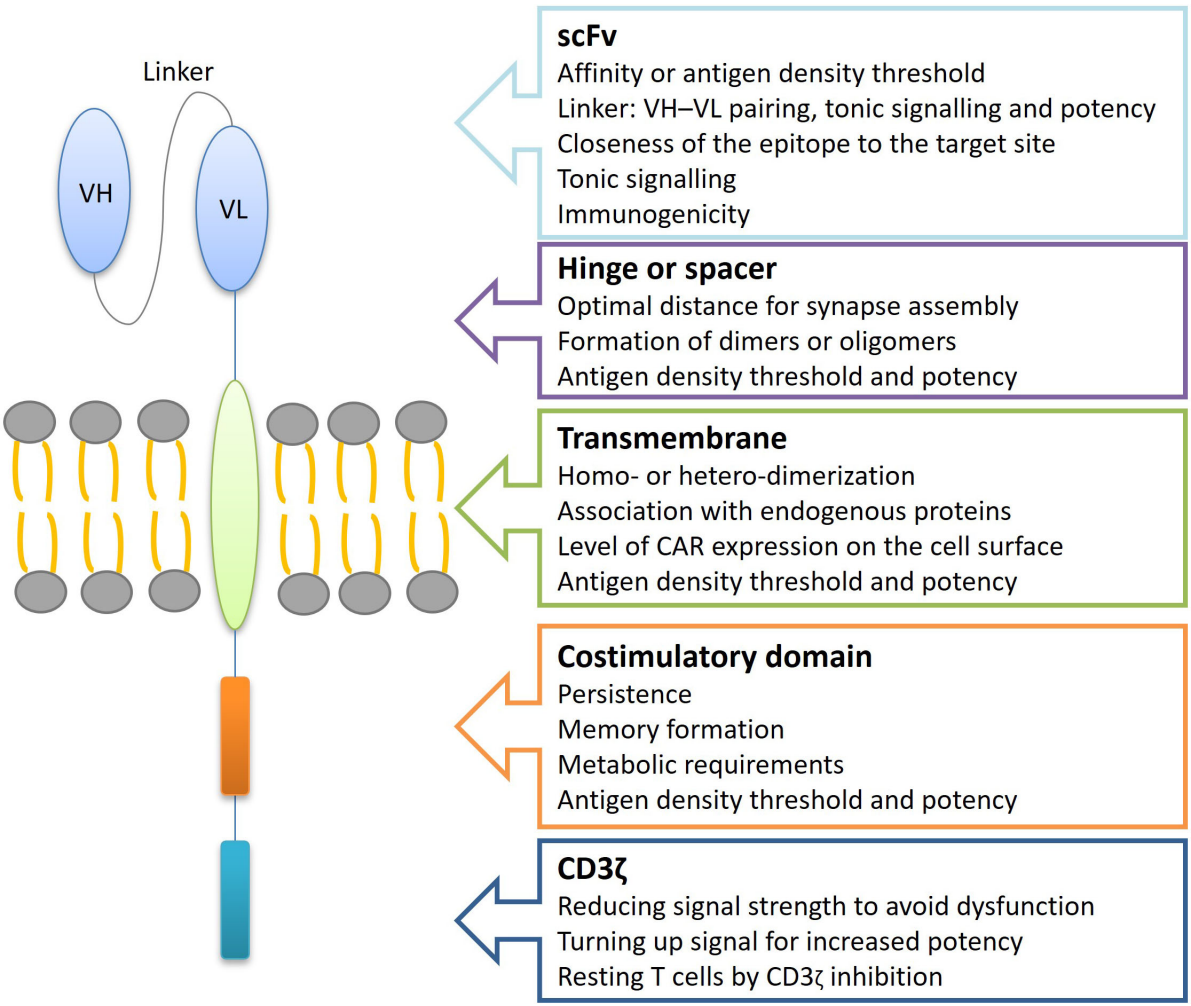
Relationship between TanCAR structure and functionality. TanCAR structure–function relationships involve understanding how changes to TanCAR components impact their performance. TanCARs consist of several key elements: two different target-binding domains like scFvs, a hinge or spacer region that extends the binding domain away from the cell surface and allows for structural flexibility, a transmembrane domain that embeds the receptor in the cell membrane, and costimulatory along with CD3ζ signaling domains responsible for transmitting activation signals. Alterations in the TanCAR structure can significantly influence TanCAR-T cell behavior, affecting factors such as the antigen density required for activation, cell persistence, efficacy, tonic signaling, TanCAR expression levels, and the likelihood of dimerization. scFv, single-chain variable fragments; VH, variable heavy chain; VL, variable light chain.

In the case of administering a single CAR-T product, one option is infusing T cells that express two different CARs in the same cell, known as bicistronic CAR-T cells. This approach uses a bicistronic vector that encodes two independent CAR molecules, separated by a ribosomal skip sequence. Alternatively, TanCAR-T cells, which encode two CARs within the same chimeric protein using a single vector, can also be infused (**Figure 3**). The tandem structure can take two forms: the classical tandem configuration, where the variable light chain (VL) and variable heavy chain (VH) sequences of one scFv are directly linked to the VL-VH sequences of a second scFv, or the loop structure, where the VL and VH sequences of one scFv are intercalated with those of the other scFv. Additionally, CAR-T cells can be bispecific or trispecific, meaning they can target two or three antigens simultaneously (26–30).

Multi-target CAR-T cells offer several advantages, including precise localization at the tumor site, high cytotoxicity towards tumor cells, reduced off-target effects, and more precise treatment (31). Pooled CAR-T cells, from co-administration, exhibit lower cytokine secretion and cytolysis compared to TanCAR-T cells and bicistronic CAR-T cells, but still higher than single CAR-T cells. It is worth noting that using two CAR-T cell lines concurrently exerts strong immune pressure on tumor cells, which may lead to the simultaneous escape of both target antigens. Besides the high cost of producing multiple vectors, the heterogeneity of the infused product can complicate clinical analysis (28). The simultaneous infusion of two CAR-T cell lines can result in an imbalance in the cell population (32). Co-administration and co-transduction methods are more expensive and labor-intensive compared to the production of bicistronic CAR-T or TanCAR-T cells (28). Preclinical studies indicate that dual-signaling CAR-T cells provide stronger anti-tumor responses than single-target CAR-T cells or mixed CAR-T products, potentially reducing recurrence due to downregulation or loss of target antigens in tumor cells (10).

Mechanisms that influence CAR T-cell efficacy are multifactorial and include tumor-related factors (e.g., antigen loss and intrinsic resistance to cytotoxicity), host-related factors, such as hostile tumor microenvironment, and product-related factors (inadequate CAR T-cells) (33). The simultaneous binding of both target antigens by TanCAR-T cells elicits an exponentially more potent response, indicating a synergistic effect of dual binding on their cytotoxic capabilities. This bivalency enhances CAR signal strength, potentially resulting in a superior antitumor functionality compared to other approaches. However, excessive CAR signal strength could lead to CAR-T cell exhaustion, reduced persistence, and an increased risk of adverse events (6). Tan-CARs have numerous therapeutic applications as they are as effective as conventional single antigen-specific CARs but are less toxic and highly effective in treating diseases with a higher frequency (3). Selecting appropriate targets is crucial to ensure that multispecific CARs are both safe and effective (26). Synergistic antitumor efficacy has been demonstrated when both antigens are simultaneously encountered. The formation of bivalent immune synapses during TanCAR-T cell exposure to tumors co-expressing both antigens is crucial for preventing antigen escape and improving antitumor efficacy (32). TanCARs demonstrate enhanced efficacy by targeting multiple antigens, allowing dual-targeted CAR-T cells to effectively recognize and eliminate tumor cells when one antigen is lost or downregulated. This improves the overall therapeutic response and reduces the risk of disease relapse and other adverse clinical outcomes. Furthermore, TanCARs also offer improved safety by requiring simultaneous engagement of both antigens for activation. This increases specificity and reduces the risk of off-target toxicities, such as cytokine release syndrome and neurological toxicity, thereby enhancing the safety profile of CAR-T cell therapy (4).

## 3 Tandem CAR-T cells: preclinical studies

There are preclinical data showing improved efficacy for dual-targeting compared to single-targeting CAR-T cells. The preclinical data on TanCAR-T cells in hematological malignancies and solid tumors are listed in **Table 1**.

**Table 1:** Published data on preclinical studies of tandem CAR-T cells in cancer.

Type of cancer	Targets	Transmembrane region	Co-stimulatory domain	Vector	Reference
MM	BCMA/CD24	CD28	4-1BB	Lentiviral	(34)
MM	BCMA/CD38	CD8	CD28	Retroviral	(35)
MM	BCMA/CD19	CD8	CD28	Lentiviral	(36)
MM	BCMA/GPRC5D	Non specified	4-1BB	Lentiviral	(37)
MM	BCMA/CS1	CD28	4-1BB	Lentiviral	(38)
T cell malignancies	CD5/CD7	CD28	4-1BB	Lentiviral	(39)
T cell malignancies	CD38/LMP1	CD28	4-1BB	Lentiviral	(40)
B cell malignancies	CD33/CLL-1	CD8	4-1BB	Lentiviral	(41)
B cell malignancies	CD123/CLL-1	CD28	OX40	Lentiviral	(42)
B cell malignancies	CD123/FRβ	CD8	4-1BB	Retroviral	(43)
B cell malignancies	CD19/CD20	CD28	4-1BB	Lentiviral	(44)
B cell malignancies	CD19/CD20	CD8	4-1BB	Lentiviral	(45)
B cell malignancies	CD19/CD20	CD8	4-1BB	Lentiviral	(46)
B cell malignancies	CD19/CD20	CD28	CD28	Retroviral	(47)
B cell malignancies	CD19/CD79	CD28	4-1BB	Lentiviral	(48)
B cell malignancies	CD19/CD79b	CD8	4-1BB	Lentiviral	(49)
B cell malignancies	CD19/CD22	CD8	4-1BB	Lentiviral	(50)
Ovarian	FOLR1/MSLN	CD8	CD28/4-1BB	Lentiviral	(15)
Ovarian	MUC16/PD-L1	CD8	CD28	Lentiviral	(17)
Lung	MUC1/PSCA	CD28	CD28	Lentiviral	(16)
Lung /melanoma	CD70/B7-H3	CD8	4-1BB	Lentiviral	(51)
Gastric	NKG2DL/CLDN18.2	CD8	4-1BB	Lentiviral	(52)
GBM	IL13Rα2/HER2	CD28	CD28	Retroviral	(53)
GBM	IL13Rα2/EGFRvIII	CD8	4-1BB	Lentiviral	(18)
GBM	IL13Rα2/EphA2	CD28	CD28/CD134	Lentiviral	(54)
GBM	IL13Rα2/TGF-β	CD8	4-1BB	Retroviral	(55)

BCMA, B cell maturation antigen; CLDN18.2, claudin18.2; CLL-1, C-type lectin-like molecule-1; EGFRvIII, epidermal growth factor variant III; EphA2, erythropoietin-producing hepatocellular carcinoma A2; FOLR1, folate receptor 1; FRβ, folate receptor beta; GBM, glioblastoma; GPRC5D, G protein-coupled receptor class-C group-5 member-D; HER2, human epidermal growth factor receptor 2; IL13Rα2, interleukin-13 receptor α2; LMP1, Latent membrane protein 1; MM, multiple myeloma; MSLN, mesothelin; MUC1, mucin 1; MUC16, mucin 16; NKG2DL, natural-killer group 2 member D ligands; PD-L1, programmed death ligand 1; PSCA, prostate stem cell antigen; TGF-β, transforming growth factor-beta.

### 3.1 Multiple myeloma

B-cell maturation antigen (BCMA) is highly expressed on the surface of MM cells, with low expression in normal cells and none in CD34^+^ hematopoietic cells (56). This selective expression has led to substantial interest in targeting BCMA for therapeutic purposes, especially given its presence in plasma cells (36). Anti-BCMA CAR-T cell therapies represent a promising treatment strategy with high response rates in MM (34). However, several clinical trials have noted patient relapses linked to downregulation or complete loss of BCMA in tumor cells, reducing CAR-T cell efficacy. To address this issue, some studies are investigating the development of TanCARs as a treatment strategy for MM. For this purpose, selecting another antigen widely expressed in MM cells is necessary (35, 57).

In the tumor microenvironment (TME), CD24 is an important checkpoint molecule regulating the innate immune response and is expressed in myeloma cells that relapse following BCMA-targeted CAR-T therapy. Consequently, CD24 may serve as a target for immunotherapy. Fumou et al. developed a dual-targeted BCMA/CD24 CAR-T therapy, comparing the efficacy of monospecific CAR-T, bicistronic CAR-T and TanCAR-T cells. However, they found that bicistronic CAR-T cells demonstrated superior *in vitro* activity over TanCAR-T cells, leading them to focus on bicistronic CAR-T cells for their *in vivo* experiments (34).

CD38 has also emerged as a promising antigen target for MM due to its high expression in MM cells, including therapy-resistant and myeloma-initiating cells. CD38-targeted CAR-T cells have shown anti-MM activity in preclinical studies (58). One study reports a TanCAR-T targeting both BCMA and CD38, which can induce robust cytotoxicity against target cells expressing either BCMA or CD38. In *in vitro* experiments, this study demonstrated that these TanCAR-T cells exhibited similar CAR expression, superior cytotoxicity, and antigen-stimulated T cell proliferation compared to monospecific CAR-T cells. Furthermore, these TanCAR-T cells achieved complete tumor clearance with no relapse observed, suggesting an effective solution to the challenge of antigen escape in BCMA CAR-T cell therapies (35).

CD19 is another potential target for MM treatment. In a patient with advanced MM treated with anti-CD19 CAR-T cells autologous stem-cell transplantation (ASCT), sustained remission was observed, highlighting its therapeutic potential. Kang et al. developed a TanCAR targeting both CD19 and BCMA antigens. They found that TanCAR-T cells targeting one or both antigens produced similar cytotoxic effects *in vitro* compared to conventional single-target CAR-T cells. Interestingly, *in vivo* studies suggested that TanCAR-T cells could eradicate mixed population of malignant cells expressing either CD19 or BCMA, leading to complete remission. These findings indicate that tandem dual-antigen targeting could offer an effective antineoplastic therapy and might prevent relapse due to the absence or loss of BCMA expression in MM cells (36).

G protein-coupled receptor class C group 5 member D (GPRC5D) has been identified as a plasma cell-specific target, making it another attractive candidate to pair with BCMA. Larrea et al. compared subtherapeutic doses of different forms of dual-targeted therapies against BCMA and GPRC5D, including pooled mono-targeted CAR-T, bicistronic CAR-T and TanCAR-T cells. They found that TanCAR-T cells were unable to induce as strong or durable response as pooled or bicistronic CAR-T cells. Although the long, flexible linker used in the TanCAR design is intended to optimize binding of different scFvs and antigens, further modifications, such as reversing the scFv order for BCMA and GPRC5D, might improve outcomes. In their study, the membrane-distal GPRC5D scFv in the TanCAR configuration was less efficacious than when positioned in its membrane-proximal location, as seen in the traditional bicistronic CAR and pooled CAR approaches (37).

CS1 (also known as SLAMF7 or CD319) is widely expressed in various MM types and has been detected in 90-97% of MM patient samples. Zah et al. proposed dual-targeting of BCMA and CS1 could enhance the effectiveness of BCMA-targeting therapies while providing a safeguard against tumor escape due to BCMA loss. Their study demonstrated that BCMA/CS1 TanCAR-T cells had superior CAR expression and functionality compared to T cells co-expressing individual BCMA and CS1 CARs. Additionally, combining CAR-T cell therapy with an anti-PD-1 antibody accelerated initial tumor clearance *in vivo*, while CAR-T cell treatment alone achieved sustained, tumor-free survival even after tumor rechallenge. These findings suggest that BCMA/CS1 bispecific CAR represents a promising strategy to prevent antigen escape in CAR-T cell therapy for MM (38).

### 3.2 T cell malignancies

Expanding the success of bispecific CAR-T therapies for treating r/r T cell leukemia and lymphoma remains challenging due to the heightened risk of CAR-T cell fratricide and potential safety concerns when targeting multiple T cell antigens. A key research focus is replacing the scFv with alternative molecules to construct bispecific CARs to overcome CAR-T therapy failure or disease relapse in T cell malignancies caused by epitope or antigen loss (39).

The expression patterns of CD5 and CD7 suggest that they are promising therapeutic targets for T cell malignancies. Dai et al. conducted a functional comparison of different bispecific CAR structures, including TanCARs and dual CARs, to identify the optimal construct for combating antigen escape in a clinical setting. In their study, they developed fratricide-resistant CD5/CD7 bispecific CAR-T cells by using CRISPR/Cas9 technology to disrupt the CD5 and CD7 genes in T cells prior to transduction. The results demonstrated that TanCARs targeting CD5 and CD7 were more effective than bicistronic CARs in preventing tumor escape and controlling leukemic cells with heterogeneous antigen expression. TanCAR-T cells eliminated neoplastic cells in mice and achieved significantly prolonged leukemia suppression compared to bicistronic CAR-T cells. Additionally, the study found that TanCAR-T cells maintained more durable *in vivo* expansion (39).

Natural killer/T cell lymphoma (NKTCL) is a highly aggressive lymphoma associated with Epstein-Barr virus (EBV) infection. Research on CAR-T cell therapy for NKTCL remains limited, though CD38-targeted CAR-T cells have been explored as potential immunotherapeutic agents. Latent membrane protein 1 (LMP1), encoded by EBV, has been shown to drive malignant transformation in B cells and epithelial cells. An *in vitro* study by Hongwen et al. with NKTCL cells revealed that CD38/LMP1 TanCAR-T cells exhibited significantly greater cytotoxicity than single-target CAR-T cells, with similar results observed in cytokine secretion. *In vivo* experiments showed that mice treated with CD38/LMP1 TanCAR-T cells had smaller tumor burdens than those treated with single-target CAR-T cells, highlighting the potential of TanCAR-T cell therapy for NKTCL (40).

### 3.3 B cell malignancies

CAR-T cell therapy has revolutionized the treatment of r/r B cell malignancies. However, challenges such as tumor antigen escape, severe cytokine release syndrome (CRS), and disease recurrence persist in some patients after treatment (42). Despite significant progress over the last decade in identifying leukemia-associated antigens as therapeutic targets, clinical results have not consistently met expectations. This may be due to the limitations of single-antigen targeting, which can fail to eliminate all AML blasts and pose a risk of fatal off-tumor, on-target side effects (41). To overcome these challenges, multi-targeted CAR-T approaches are being developed as alternatives to currently approved CAR-T therapies.

Following the success of CAR-T cell therapy in treating B cell hematologic malignancies, researchers are now exploring its application in AML, targeting antigens such as interleukin-3 receptor α (CD123), folate receptor beta (FRβ), CD33, Lewis Y, C-type lectin-like molecule-1 (CLL-1), NKG2D, and CD44v6 (42, 43).

Given the therapeutic potential of targeting both CD33 and CLL-1 in AML, dual-targeting CD33/CLL-1 CAR-T cells hold significant promise. Wang et al. constructed a CD33/CLL-1 TanCAR that demonstrated potent cytotoxicity against leukemia cell lines and primary AML cells *in vitro*. Co-culture of AML blasts with TanCAR-T cells led to robust proliferation and high levels of granulocyte-macrophage colony-stimulating factor (GM-CSF) and interferon-γ (IFN-γ) secretion. Notably, TanCAR-T cells showed minimal impact on normal hematopoietic stem cells (HSCs), supporting safety *in vivo*. In mouse models, these cells exhibited significant anti-leukemic activity, leading to tumor eradication and prolonged survival. These findings underscore the clinical potential of CD33/CLL-1 TanCAR-T cells as a safe and efficient therapeutic strategy for AML, potentially reducing the need for transplantation (41).

CD123 is a critical target expressed on CD19-negative blasts that relapse after CAR-T cell therapy. However, previous studies have shown that CD123-targeting agents alone have limited efficacy against leukemia cells (43). CD123 and CLL-1 are not only expressed in most leukemia cells but also highly expressed in leukemia stem cells (LSCs), making them promising targets for AML treatment. Wang et al. developed TanCAR-T cells targeting CD123 and CCL-1, which exhibited significant cytotoxic effects on CLL-1^+^CD123^+^ leukemia cell lines and primary AML cells. These *in vitro* experiments suggest that dual-target CAR-T cells broaden the therapeutic range compared to single-target CAR-T cells and enhance anti-tumor activity (42).

Another promising target is FRβ, expressed in approximately 70% of AML cases, with limited expression in normal tissues but high expression in B-AML blasts. A study demonstrated that TanCAR-T cells targeting FRβ and CD123 effectively lysed leukemia cell lines, similar to single CAR-T cells targeting these antigens individually. Moreover, the bispecific interaction resulted in significantly higher cytokine secretion by TanCAR-T cells compared to monospecific CAR-T cells. While exposure to a single antigen causes a decline in cytokine release at certain antigen densities in monospecific CAR-T cells, TanCAR-T cells maintained elevated cytokine levels, demonstrating enhanced functionality (43).

CD19 CAR-T cell therapy has achieved remarkable clinical outcomes in treating acute and chronic B cell malignancies, showing curative potential for relapsed B cell malignancies. However, these trials have also exposed vulnerabilities in current CAR technology, including susceptibility of tumor cells to antigen escape (44). Despite the overall success of CD19 CAR-T therapy, reports are emerging of patients relapsing with CD19-negative disease following CD19-directed treatment (45, 47). This presents a significant challenge for CD19-based therapies, emphasizing the importance of targeting additional antigens (49).

Given the ubiquitous expression of CD19 and CD20 on B cells, the simultaneous loss of both antigens is considered highly unlikely. Therefore, dual targeting of CD19 and CD20 offers a promising strategy to counter antigen escape in malignant B cells (44). The results obtained by Zah et al. from their *in vitro* and *in vivo* characterization experiments indicate that TanCAR targeting both CD19 and CD20 can prevent antigen escape by targeting malignant B cells through CD20 when CD19 expression is lost. Their findings demonstrate that optimized bispecific CARs can effectively control both wild-type B-cell lymphoma and CD19^–^ mutants with equal efficiency *in vivo* (44).

Another study using TanCAR-T cells targeting CD19 and CD20 demonstrated that these tandem constructs efficiently killed the Raji cell line both *in vitro* and *in vivo*. Although TanCARs generated fewer cytokines compared to CD20 CARs, their cytokine levels were comparable to CD19 CARs. In co-culture experiments at low effector-to-target ratios, rapid down-modulation of full-length CD19 expression was observed, as well as partial down-modulation of CD22 and, to a lesser extent, CD20. The data also emphasize the extreme sensitivity of the NALM-6 cell line to general lymphocyte-mediated cytotoxicity. Under high-disease burden conditions, the TanCAR-T cells demonstrated superior efficacy and were less toxic than a mixture of transduced T cell populations expressing single CARs (45).

Chen et al. constructed TanCAR and loop CAR targeting CD19 and CD20 to assess their killing efficiency and therapeutic efficacy. Among the four bispecific CAR-T constructs tested, loop2019 CAR-T was the most effective in eradicating lymphoma cell lines and primary lymphoma or chronic lymphocytic *leukemia* (CLL) cells from patient samples at very low dose *in vitro*. In a xenograft NOD/SCID lymphoma mouse model, loop2019 CAR-T significantly prolonged mouse survival, highlighting its potential as a new treatment option for B cell malignancies, particularly CLL and lymphoma (46).

Martyniszyn et al. evaluated a TanCAR targeting CD19 and CD20, which effectively induced cell death in CD19^+^CD20^+^ CLL cells *in vitro*. When compared, both the bispecific CD20/CD19 CAR-T cells and monospecific CAR-T cells eliminated cells of the established Raji lymphoma line. In the context of heterogeneous leukemic cell populations, the bispecific CAR-T cells demonstrated an advantage by efficiently targeting CD19^+^CD20^-^ ALL cells. These findings highlights the potential of TanCAR-T cells targeting both CD19 and CD20 for treating B-cell malignancies with a high probability of relapse over the long term (47).

CD79a and CD79b, components of the B-cell receptor (BCR) signaling complex, represent new targets for the treatment of B cell lymphoma (49). In total, 87.3% of CD79ab-positive tumors also co-expressed CD19. Leung et al. examined the strengths and limitations of targeting CD19 and CD79 with bispecific CAR-T cells. They developed tandem, bicistronic, and pooled products of CD79a/CD19 or CD79b/CD19 CAR-T cells. Data showed a trend toward better tumor control with tandem and bicistronic CAR-T cells. However, when tumor cells expressed only a single antigen (i.e., CD19 or CD79), bispecific CAR-T cells showed compromised efficacy compared to monospecific CAR-T cells, due to less efficient antigen binding and reduced downstream signaling. Thus, the added benefit of dual specificity comes at the expense of reduced sensitivity to each individual target antigen (48). Another study showed that TanCAR-T cells targeting CD79b/CD19 (but not targeting CD19/CD79b in the reverse order) successfully lysed CD19^-^ lymphoma within a heterogeneous tumor. These CAR-T cells eliminated CD19^+^, CD19^-^, and mixed CD19-expressing lymphomas in xenograft models of lymphoma. These results suggest that TanCAR-T cells targeting CD79b and CD19 have the potential to treat both CD19^-^ relapsed tumors and tumors that retain CD19 expression (49).

CD22, a pan-B cell antigen, has also shown promise in CAR-T strategies, though relapse due to CD22 loss or reduced surface expression has been observed. Qin et al. developed a CD19/CD22 CAR construct, based on two clinically validated single-CAR constructs. The optimized CAR construct induced comparable levels of IFN-γ and interleukin-2 (IL-2) *in vitro* compared to single CARs when tested against dual-antigen-expressing and single-antigen-expressing cell lines. T cells expressing the CD19/CD22 CAR eradicated xenografts from ALL cell lines and patient-derived xenografts (PDX), including a PDX generated from a patient who relapsed with CD19^-^ disease following CD19-directed CAR therapy. This bivalent CAR offers an opportunity to reduce the risk of antigen loss and improve therapeutic durability (50).

### 3.4 Ovarian cancer

The antigens folate receptor 1 (FOLR1) and mesothelin (MSLN) are specifically and highly expressed in cancer tissues, with only 11.25% of samples negative for both antigens (15). Studies have shown that targeting FOLR1 or MSLN individually results in a 48-76% likelihood of near-complete tumor elimination, while simultaneous targeting of both proteins increases tumor cell killing to 88% in ovarian cancer (12). Liang et al. designed a novel third-generation TanCAR targeting both FOLR1 and MSLN, combined with IL-12 secretion, to reduce the likelihood of tumor antigen escape and enhance CAR-T cell infiltration and antitumor activity. TanCAR-T cells efficiently lysed antigen-positive ovarian cancer cells *in vitro* and secreted higher cytokines levels than single-target CAR-T cells. More importantly, *in vivo* experiments demonstrated that TanCAR-T cells significantly reduced tumor volume, exhibited enhanced antitumor activity, and prolonged survival in mouse models (15).

Other studies have shown that 80% of epithelial ovarian cancer express mucin 16 (MUC16), whose extracellular segment is cleaved and released into the peripheral blood, serving as a well-known tumor marker. Additionally, the programmed cell death protein-1 (PD-1) and its ligand, programmed death-ligand 1 (PD-L1), form the PD-1/PD-L1 signaling pathway, which plays an inhibitory role in T cell immunity. Therefore, it is reasonable to speculate that a TanCAR combining PD-1 and anti-MUC16 could be an effective strategy against epithelial ovarian cancer. Li et al. developed TanCAR-T cells targeting PD-L1 and MUC16. Although no significant differences were observed between TanCAR-T cells and single-target CAR-T cells in cytotoxicity and cytokine production against OVCAR-3 cells *in vitro*, TanCAR-T cells demonstrated a remarkable therapeutic effect *in vivo*, prolonging survival in tumor-bearing mouse models compared to single CAR-T cells. These findings suggest that TanCAR-T cells hold promising therapeutic potential, and the experimental data may support further research leading to clinical studies (17).

### 3.5 Lung cancer

Mucin 1 (MUC1) and prostate stem cell antigen (PSCA) are overexpressed in non-small cell lung cancer (NSCLC). Abnormal glycosylation in MUC1 commonly occurs in many epithelial cancers, including NSCLC. High MUC1 expression has been observed in 86.3% of lung adenocarcinoma cases and 39.1% of lung squamous cell carcinoma cases. Immunohistochemical staining of NSCLC tissue after surgery has been revealed to have a high PSCA expression in 68.8% of tumor tissues. Additionally, high MUC1 and PSCA expression in patients is often associated with a poor prognosis. Therefore, MUC1 and PSCA show potential as targets for lung cancer diagnosis and immunotherapy. Results obtained by Wang et al. indicate that TanCAR-T cells targeting both MUC1 and PSCA have a more effective tumor-killing effect than single-target CAR-T cells, with their antitumor efficacy further enhanced by anti-PD-1 antibody treatment. This study reports a previously unexamined therapeutic effect of TanCAR-T cells in NSCLC, providing a preclinical rationale for combining anti-PD-1 antibodies with TanCAR-T cells targeting MUC1 and PSCA in NSCLC treatment (16).

CD70, the ligand of CD27, exhibits aberrant expression in hematological malignancies and in some solid tumors. Several reports have provided evidence suggesting the therapeutic potential of CD70 CAR-T cells. In addition, B7-H3 (CD276), a type I transmembrane protein, is generally expressed at low levels in normal tissues but is overexpressed in a wide variety of cancers, including gastric cancer, pancreatic cancer, neuroblastoma, endometrial cancer, glioma, melanoma, lung cancer, ovarian carcinomas, and prostate cancer, positioning B7-H3 as a promising immunotherapeutic target. In a study by Yang et al., TanCAR-T cells targeting both CD70 and B7-H3 demonstrated enhanced antitumor functionality and addressed the issue of antigenic heterogeneity in lung cancer treatment. TanCAR-T cells showed increased cytolytic activity and cytokine release compared to monospecific CAR-T cells when encountering tumor cells expressing two target antigens. Furthermore, low doses of TanCAR-T cells effectively controlled lung cancer xenografts and improved overall survival (OS) in treated animals (51).

### 3.6 Melanoma

The TanCAR targeting CD70 and B7-H3, developed by Yang et al. for the treatment of lung cancer, was also validated in melanoma through both *in vitro* and *in vivo* studies. In a melanoma mouse model generated by injecting the A375 melanoma cell line, treatment with TanCAR-T cells led to a more pronounced reduction in tumor burden compared to controls and the single-target CAR (CD70 or B7-H3) groups. The results observed in melanoma were consistent with those observed in lung cancer, further supporting the efficacy of this dual-targeting approach (51).

### 3.7 Gastric cancer

It has been reported that claudin18.2 (CLDN18.2) is the most promising target for gastric cancer. Xu et al. constructed novel TanCAR-T cells, named KD-496, which simultaneously recognize NKG2D ligands (NKG2DL) and CLDN18.2. These KD-496 CAR-T cells demonstrated superior antitumor efficacy and safety *in vitro* and *in vivo* compared to monospecific CAR-T cells. These TanCAR-T cells showed antigen-specific activation though cytokine secretion and tumor cell cytotoxicity assays. When co-incubated with gastric cancer cells, NUGC4 and MKN-28-18.2, KD-496 CAR-T cells specifically upregulated IFN-γ and strongly lysed tumor cells, even at low effector-to-target ratios. Remarkably, *in vivo* studies showed that KD-496 CAR-T cells more efficiently eliminated xenograft tumors and did not exhibit significant treatment-related toxicity in treated mice. These results support the potential for future clinical trials of KD-496 TanCAR in patients with gastric cancer, where the need for effective treatment is substantial (52).

### 3.8 Glioblastoma

CAR-T cell therapy for glioblastoma (GBM) targets several antigens, including interleukin-13 receptor α2 (IL13Rα2), human epidermal growth factor receptor 2 (HER2), epidermal growth factor variant III (EGFRvIII), and erythropoietin-producing hepatocellular carcinoma A2 (EphA2) (18, 54). Among these, IL13Rα2 is considered the most suitable target for GBM, as it is overexpressed in approximately 50-80% of gliomas, while remaining undetectable in normal brain tissue (54, 55). In preclinical models of GBM, CAR-T cells have demonstrated robust antitumor activity and are currently being evaluated in phase I/II studies targeting glioma-specific, antigens IL-13Rα2, HER2, and EGFR. A mathematical model analyzing the expression hierarchy of three validated glioma antigens (HER2, IL13Rα2, and EphA2) predicted an increased likelihood of tumor elimination when any two of these three antigens are targeted (59).

Hedge et al. developed a TanCAR specific to two glioma-associated antigens, HER2 and IL13Rα2, and found that TanCAR-T cells could simultaneously engage both antigens by inducing HER2-IL13Rα2 heterodimers, which promoted superadditive T cell activation when both antigens were encountered concurrently. TanCAR-T cell demonstrated more sustained activity without increased susceptibility to exhaustion, compared to T cells that co-expressed HER2 and IL13Rα2 CARs, monospecific CAR-T cells, or pooled products. In a murine GBM model, TanCAR-T cells mitigated antigen escape, exhibited enhanced antitumor efficacy, and improved animal survival. These findings suggest that TanCAR-T cells have therapeutic potential to improve GBM control by co-engaging HER2 and IL13Rα2 in an enhanced, bivalent immune synapse that strengthens T cell functionality and reduces antigen escape (53). However, HER2 scFv-based CAR-T cells can harm normal cells due to off-target recognition of HER2, which are also expressed in some healthy cells (54).

In contrast to other GBM-associated surface antigens, such as HER2, EGFRvIII and IL-13Rα2 are unique because they are frequently expressed in GBM cells but are absent or present at very low levels in somatic tissues, reducing the likelihood of on-target, off-tumor toxicity. Recent studies indicate that IL-13Rα2 and EGFRvIII expression is significantly heterogeneous in glioma tissues, suggesting that strategies targeting both antigens simultaneously could achieve broader tumor coverage. Schimdt et al. developed TanCAR-T cells targeting both EGFRvIII and IL-13Rα2, which showed superior cytotoxicity *in vitro* against heterogeneous GBM populations, including patient-derived tumor cultures. TanCAR-T cells also exhibited faster and more complete cytotoxicity compared to monospecific CAR-T cells, and importantly, did not exhibit higher off-target effects than CAR-T cells targeting only EGFRvIII. In mouse models of GBM, including PDX models, TanCAR-T cells led to complete and durable responses, demonstrating superior activity against heterogeneous tumors compared to their monospecific CAR counterparts (18).

EphA2 is also overexpressed in GBM and, unlike EGFRvIII, is not associated with the development of antigen loss variants, making it a safer target for CAR therapy. Consequently, EphA2 represents another promising target for GBM treatment. Muchammad et al. developed a TanCAR that targets both IL13Rα2 and EphA2, with the potential to more selectively kill heterogeneous gliomas and address antigen escape in CAR-T cell therapy for GBM. The third-generation TanCAR-T cells exhibit more selective killing of heterogeneous GBM, with a reduced likelihood of antigen escape when both targets are encountered and can enhance the persistence of CAR-T cells at the tumor site. This novel TanCAR demonstrates superior antitumor activity compared to single-specificity CARs. Additionally, the bispecific TanCAR has the potential to reduce off-target cytotoxicity and mitigate tumor antigen escape (54).

Transforming growth factor-beta (TGF-β), commonly overexpressed in solid tumors, plays a significant role in the GBM TME by promoting the tumorigenicity of glioma-initiating stem cells, tumor-cell proliferation, and invasiveness. Additionally, TGF-β modulates immune cell composition and function, facilitating tumor evasion of immune responses and making it a promising therapeutic target. A recent study engineered a TanCAR targeting both IL-13Rα2 and TGF-β, designed to reprogram tumor-specific T cells to convert TGF-β from an immunosuppressant to an immunostimulant. These TanCAR-T cells were evaluated for efficacy and safety in both patient-derived GBM xenografts and syngeneic murine glioma models. Treatment with IL-13Rα2/TGF-β CAR-T cells resulted in increased T-cell infiltration and reduced levels of suppressive myeloid cells in the tumor-bearing brain compared to conventional IL-13Rα2 CAR-T cells, leading to improved survival in both patient-derived GBM xenografts and syngeneic murine models. These findings demonstrate that by reprogramming tumor-specific T-cell responses to TGF-β, bispecific IL-13Rα2/TGF-β CAR-T cells can counter and remodel the immunosuppressive TME, driving potent anti-tumor responses in GBM (55).

## 4 Tandem CAR-T cells: clinical trials

Clinical studies employing TanCAR-T cells are still limited and are currently in early clinical stages, with only a few studies providing preliminary data. **Table 2** presents TanCAR strategies that have been translated into sixteen clinical trials targeting various antigen combinations, including BCMA/CD38, BCMA/CD19, BCM/CS1, CD19/CD20, CD19/CD22 and NKG2DL/CLDN18.2. More research and clinical trials are still needed to improve the effectiveness and safety of these therapies and determine the best ways to combine them with other treatments. CAR-T cell therapies represent some of the most advanced cancer treatments available today. Their development and success have led to FDA approval for the treatment of blood cancers, while research is ongoing to expand their use for solid tumors. The role of tandem CAR-T cell therapy in current treatment algorithms must be carefully considered before implementing it in clinical practice. Tandem CAR-T cells may be used as a rescue treatment for hematologic malignancies when single-target CAR-T treatments have failed because of antigen escape, or as a frontline therapy for patients with high-risk disease characteristics. To improve overall treatment efficacy, tandem CAR-T therapy may be used in solid tumors in conjunction with other modalities like checkpoint inhibitors or traditional chemotherapy. Many targeted therapies are becoming standard treatment for cancer. However, success rates can vary depending on multiple factors, including the type of cancer and the individual’s response to therapy (1). There have been 16 clinical trials investigating TanCAR-T cells with varying statuses: prospective registration (n = 1), recruiting (n = 5), active but not recruiting (n = 2), completed (n = 3), and studies with unknown status (n = 4) (www.clinicaltrials.gov). **Table 3** provides a summary of the clinical data from these clinical trials, including their results.

**Table 2:** Published data of tandem CAR-T cells clinical trials.

Study Title	Clinical trial identifier	Targets	Phase	Status
BCMA-CD38 Bispecific CAR-Modified T Cells in the Treatment of r/r MM	ChiCTR1800018143	BCMA CD38	1	Recruiting
Phase I/II Study to Evaluate Treatment of r/r MM With Dual CAR-T Cells Targeting CD38 and BCMA	NCT03767751	BCMA CD38	1/2	Unknown status
Clinical study for anti-BCMA and anti-CD19 double targets CAR-T in the treatment of r/r plasma cell tumor	ChiCTR2000033567	BCMA CD19	1/2	Recruiting
Safety and Efficacy of the Bispecific CAR T Therapy Targeting CS1 and BCMA in Patients With r/r MM: a Single-center, Open-label, Single-arm Clinical Study	NCT04662099	BCMA CS1	1	Recruiting
Phase 1/1b Study of Redirected Autologous T Cells Engineered to Contain an Anti CD19 and Anti CD20 scFv Coupled to CD3ζ and 4-1BB Signaling Domains in Patients With r/r CD19 or CD20 Positive B-Cell Malignancies	NCT03019055	CD19 CD20	1	Completed
Phase I/II Study of Tandem, Bispecific Anti-CD19 Anti-CD20 CAR-T Cells for Patients With r/r B-Cell Malignancies	NCT04186520	CD19 CD20	1/2	Recruiting
A Phase I/II Safety, Dose Finding and Feasibility Trial of MB-CART2019.1 in Patients With Relapsed or Resistant CD20 and CD19 Positive B-NHL	NCT03870945	CD19 CD20	1/2	Completed
A Pivotal Phase II Randomised, Multi-centre, Open-label Study to Evaluate the Efficacy and Safety of MB-CART2019.1 Compared to SoC Therapy in Participants With r/r DLBCL, Who Are Not Eligible for HDC and ASCT	NCT04844866	CD19 CD20	2	Active, not recruiting
A Multi-center Single Arm Phase II Study to Evaluate the Safety and Efficacy of Genetically Engineered Autologous Cells Expressing Anti-CD20 and Anti-CD19 Specific CAR in Subjects With r/r DLBCL	NCT04792489	CD19 CD20	2	Recruiting
Clinical Study of CD19/CD20 tanCAR T Cells in Relapsed and/or Chemotherapy Refractory B-cell Leukemias and Lymphomas	NCT03097770	CD19 CD20	1/2	Completed
Clinical Trial of CD19/CD20 Dual Target CAR-T Cells in the Treatment of r/r B-cell Lymphoma	NCT04723914	CD19 CD20	1/2	Unknown status
Clinical Study of CD19/CD22 Tan CAR T Cells in Relapsed and/or Chemotherapy Refractory B-cell Leukemias and Lymphomas	NCT03185494	CD19 CD22	1/2	Unknown status
Phase 1 Dose Escalation Study of CD19/CD22 CAR T Cells With or Without NKTR-255 in Adults With Recurrent or Refractory B Cell Malignancies	NCT03233854	CD19 CD22	1	Active, not recruiting
Safety and Efficacy of CAR T Cell Treating r/r CD19/CD20/CD22/CD30 Postive NHL	NCT03196830	CD19 CD22	2	Unknown status
Safety and efficacy of dual CD19/CD22 targeted CAR T cells for hematological malignancies	ChiCTR1800015575	CD19 CD22	1	Prospective registration
A Single-center, Open-label, Single-arm Clinical Study of the Safety and Efficacy of KD-496 CAR-T Therapy in Advanced NKG2DL+/CLDN18.2+ Solid Tumors	NCT05583201	NKG2DL CLDN18.2	1	Recruiting

ASCT, autologous stem-cell transplantation; BCMA, B-cell maturation antigen; B-NHL, B-cell Non Hodgkin Lymphoma; CAR, chimeric antigen receptor; CLDN18.2, claudin18.2; DLBCL, diffuse large B cell lymphoma; HDC, high dose chemotherapy; MM, multiple myeloma; NHL, non-Hodgkin lymphoma; NKG2DL, natural-killer group 2 member D ligands; SoC, standard of care.

**Table 3:** Published results of efficacy and safety of TanCAR-T cells in clinical trials and preclinical studies.

Disease and target	Trial number, number of patients (pts), age, and clinical results	Preclinical results	Ref.
MM BCMA/CD38	ChiCTR1800018143 (23 pts, 59 years) ORR 87%, CR 52%, PFS 17.2 months, CRS 87% (grade 1-2 65%)	*In vitro*: specificity and activity against BCMA+ or CD38+ tumor cells. *In vivo* (xenograft mouse model): effectively eradicated MM cells	(58, 60)
MM BCMA/CD38	NCT03767751 (80 pts) N/A	N/A	(61)
MM BCMA/CD38	ChiCTR2000033567 (50 pts, 57 years) ORR 92%, PFS 19.7 months, 1-year OS rate 85%, CRS 92% (grade ≥ 3 8%), neurotoxic events (grade 1) 4%	*In vitro*: antigen-specific antitumor activity. *In vivo* (xenograft mouse models): potent antigen-specific anti-tumor activity	(62)
MM BCMA/CS1	NCT04662099 (16 pts, 60 years) ORR 81%, 1-year PFS rate 56.2%, 1-year OS rate 72.7%, CRS 38% (grade 1-2 31%)	N/A	(63)
NHL, CLL CD19/CD20	NCT03019055 (22 pts, 57 years) ORR 88% (CR 75%), 2-year PFS rate 44%, 2-year OS rate 69%, CRS (grade 3-4) 5%, neurotoxicity (grade 3-4) 14%	N/A	(64, 65)
CLL, RT CD19/CD20	NCT04186520 (14 pts, 65 years) ORR 92% (CR 46%), CRS 100%, ICANS 21%, ICE-HS 64%, deaths 1	N/A	(66)
NHL CD19/CD20	NCT04186520 (22 pts, 63 years) ORR 91% (CR 55%), CRS 86%, ICANS 27%	N/A	(67)
MCL CD19/CD20	NCT04186520 (14 pts, 63 years) ORR 100% (CR 92%), CRS (grade 1-2) 93%, ICANS 21%	N/A	(68)
NHL CD19/CD20	NCT03870945 (12 pts, 72 years) ORR 75%, CRS (grade 1-2) 66.6%, neurotoxicity 8.3%	N/A	(69)
DLBCL CD19/CD20	NCT04844866 (168 pts) N/A	N/A	(70)
DLBCL CD19/CD20	NCT04792489 (28 pts) N/A	N/A	(71)
NHL CD19/CD20	NCT03097770 (33 pts, 76% < 60 years) ORR 78%, PFS 27.6 months, CRS 70% (grade ≥3 10%), CRES (grade 3) 2%, deaths 3	*In vitro*: reduced production of cytokines (IFN-γ, TNF-α, IL-2) but more potent antitumor activity than single-targeted CAR-T cells or other TanCAR-T cells. *In vivo* (xenograft NSG mice model): antitumor potential superior to that of single-targeted CAR-T cells.	(13, 72)
NHL CD19/CD20	NCT04723914 (20 pts, 59 years) ORR 90% (CR 70%), 1-year PFS rate 60%, CRS 45%, ICANS 18%, deaths 3	*In vitro*: comparable cytotoxicity to single-targeted CAR and strong induction of cytokines in the presence of target tumor cells	(14)
B-ALL CD19/CD22	NCT03185494 (6 pts, 27.8 years) MRD-negative CR 100%, CRS 100% (grade 1-2)	*In vitro*: strong induction of cytokines in the presence of tumor target	(73)
B-ALL, DLBCL CD19/CD22	NCT03233854 (B-ALL: 17 pts, 47 years, DLBCL: 22 pts, 69 years) MRD− CR 82% (B-ALL), ORR 62% (DLBCL), PFS 3.2 months, CRS 76%, neurological toxicity 37%	*In vitro*: TanCAR-T cells secrete less cytokine when stimulated through the CD22 scFv	(74)
B-NHL CD19/CD22	NCT03196830 (32 pts, 75%<60 years) ORR 79.3% (CR 34.5%), 1-year PFS rate 40%, 1-year OS rate 63.3%, CRS 90.6% (grade≥3 28.1%), neurologic events 15.6% (grade≥3 12.5%), deaths 1	N/A	(75)
B-ALL CD19/CD22	ChiCTR1800015575 (16 pts, 27 years) ORR 87.5% (CR 62.5%), 2-year PFS 40.2%, 2-year OS rate 77.3%, CRS 100%	*In vitro*: in the presence of both antigens tended to produce more granzyme B, higher degree of cytotoxicity when compared with the monospecific CAR-T cells. *In vivo* (xenograft animal model): effectively eradicating Nalm6- GFPluc cells	(76, 77)
B-NHL CD19/CD22	NCT03196830 (33 pts, 55 years) ORR 90.9% (CR 63.6%), 2-year OS 54.3%, 2-year PFS rate 47.2%, CRS 75.8% (grade ≥3 21.2%), ICANS 9.1%	N/A	(78)
Gatric cancer NKG2DL/ CLDN18.2	NCT05583201 (18 pts) N/A	*In vivo* (PDX model): more efficient tumor elimination than single-target CAR-T cells with a favorable safety profile	(79)

B-ALL, B-cell acute lymphocytic leukemia; B-NHL, B-cell Non Hodgkin Lymphoma; CLDN18.2, claudin18.2; CLL, chronic lymphocytic leukemia; CRES, CAR T-cell-related encephalopathy syndrome; CRR, complete remission rate; CRS, cytokine release syndrome; DLBCL, diffuse large B cell lymphoma; ICANS, immune effector cell–associated; IFN- γ, interferon-γ; IL-2, interleukin-2; MRD, minimal residual disease; MCL, mantle cell lymphoma; MM, multiple myeloma; NA, not applicable; NHL, non-Hodgkin lymphoma; NKG2DL; natural-killer group 2 member D ligands; ORR, overall response rate; OS, overall survival; pts, patients; PDX, patient-derived xenografts; PFS, progression-free survival; RT, Richter’s Transformation; TNF-α, tumor necrosis factor-α.

### 4.1 Multiple myeloma

#### 4.1.1 TanCARs targeting BCMA and CD38

Mei et al. constructed a humanized TanCAR targeting both BCMA and CD38 (BM38 CAR) and evaluated its anti-myeloma activity both *in vitro* and *in vivo*. BM38 CAR-T cells demonstrated enhanced cytotoxicity *in vitro* and showed potent anti-myeloma activity in xenograft mouse models. Subsequently, twenty-three patients with r/r MM received BM38 CAR-T cell infusions in a phase 1 dose-escalation and expansion study (ChiCTR1800018143). Notably, BM38 CAR-T cells were detectable in 77.8% of evaluable patients at 9 months and in 62.2% at 12 months. This study was the first to demonstrate that humanized bispecific BM38 CAR-T cells are feasible, safe and significantly effective in patients with r/r MM, showing substantial *in vivo* persistence, deep and durable responses, and effective elimination of extramedullary disease (58). The TanCAR achieved an overall response rate (ORR) of 87%, with minimal residual disease negativity in 87.5% of cases and a median progression-free survival (PFS) of 17.2 months (60). These preliminary results require confirmation in future multicenter clinical trials.

There is another study examining tandem CD38/BCMA targeting CAR-T cells in r/r MM (NCT03767751) (61). It is a phase 1/2 study aiming to determine the safety of dual-specificity CD38 and BCMA CAR-T cells, as well as the feasibility of producing enough cells to treat patients with r/r MM. However, to date, no data has been published regarding the results of this clinical trial.

#### 4.1.2 TanCARs targeting BCMA and CD19

Plasma cells can lose CD19 antigen expression during differentiation; however, neoplastic plasma cells may retain this expression, supporting the potential use of anti-CD19 CAR-T cell therapy in MM. Consequently, bispecific CAR-T cells targeting both BCMA and CD19 have been developed to enhance tumor cell killing (60). In one study, a TanCAR-T targeting BCMA and CD19 (BC19 CAR) was designed and tested for its anti-myeloma activity *in vitro* and *in vivo*. Preclinical results indicated that BC19 CAR-T cells selectively killed cancer cells expressing either BCMA or CD19 and demonstrated potent antigen-specific anti-tumor activity in xenograft mouse models. Based on these preliminary findings, an open-label, single-arm, phase 1/2 study of BC19 CAR-T cells was conducted in 50 patients with r/r MM (ChiCTR2000033567). BC19 CAR-T cells were well tolerated and showed significant clinical efficacy, with grade 3 or higher CRS observed in 8% of patients and grade 1 neurotoxic events in 4% of patients. The ORR in this population was 92% with a median PFS of 19.7 months and a one-year OS rate of 85%. Overall, the study reported that BC19 bispecific CAR-T cell infusion is a viable, safe, and effective strategy for managing patients with r/r MM (62).

#### 4.1.3 TanCARs targeting BCMA and CS1

CS1 is highly expressed in MM cells both at diagnosis and relapse stages, and anti-CS1 therapy with elotuzumab, is approved for the treatment of r/r MM. Irreversible loss of BCMA has been observed in some relapsed patients after BCMA-targeted CAR-T cell infusion, although their MM cells retained CS1 expression. To enhance targeting of BCMA along with CS1, Li et al. developed a tandem bispecific CS1/BCMA CAR containing a novel anti-CS1 scFv (clone 7A8D5) and a novel anti-BCMA scFv (clone 4C8A). They reported preliminary data on the safety, efficacy, and kinetics of tandem CS1/BCMA CAR-T cells in 16 patients with r/r MM in a phase 1 clinical trial (NCT04662099). Among the responders, the ORR was 81%, with one year OS and PFS rates of 72.73% and 56.26%, respectively. Four patients experienced BCMA^+^ and CS1^+^ relapse or progression, and one patient responded after treatment failure with anti-BCMA CAR-T cells. These results suggest that tandem CS1/BCMA CAR-T cells have clinical activity and a favorable safety profile in patients with r/r MM (63).

### 4.2 B cell malignancies

#### 4.2.1 TanCARs targeting CD19 and CD20

CAR-T cells targeting CD19 represent a breakthrough treatment for r/r B cell malignancies. However, despite impressive outcomes, relapse with CD19-negative disease remains a challenge. Shah et al. addressed this limitation in a first-in-human trial of bispecific CD20/CD19 (LV20.19) CAR-T cells for r/r B cell malignancies. Twenty-two adult patients with B cell non-Hodgkin lymphoma (NHL) or CLL were treated in a phase 1 dose-escalation and expansion trial (NCT03019055) to evaluate the safety of TanCAR-T cells and the feasibility of on-site manufacturing using the CliniMACS Prodigy system. Treatment at the target dose of 2.5 × 10^6^ cells/kg (n = 16) resulted in an ORR of 88%, with a complete response (CR) rate of 75%, on day 28. Notably, CD19 antigen loss was not observed in patients who relapsed or experienced treatment failure. The study concluded that on-site manufacturing and infusion of non-cryopreserved LV20.19 CAR-T cells is feasible, safe and highly effective with low toxicity. Bispecific CARs may enhance clinical responses by reducing the likelihood of relapse through target antigen downregulation (64). Subsequently, Shah et al. reported two-year survival outcomes and identified predictors of early response, late relapse, and survival in patients treated at the target dose (2.5 × 10^6^ cells/kg, n = 16). This trial of LV20.19 CAR-T demonstrates promising long-term outcomes for patients with r/r B cell malignancies. Bispecific LV20.19 CAR-T cells achieve high response rates in B-cell NHL and CLL, with two-year PFS and OS rates of 44% and 69%, respectively, across all patients, and 50% and 75%, respectively, in CAR-naïve diffuse large B-cell lymphoma (DLBCL) patients. As with other CARs constructs, the efficacy of the LV20.19 CAR was impacted by high metabolic tumor volume (65).

To address the limitations observed and improve clinical outcomes, a new trial with LV20.19 CAR T-cells in r/r NHL (NCT04186520) was designed incorporating key modifications in lymphodepletion and manufacturing. As part of the ongoing phase 1/2 multi-cohort trial, fourteen patients with r/r Richter’s Transformation and CLL received LV20.19 CAR-T cells, with initial safety and outcomes reported. Although LV20.19 CAR-T cells demonstrated efficacy in both CLL and RT, their use was limited by high rates of immune effector cell-associated hemophagocytic lymphohistiocytosis (IEC-HS), particularly among CLL patients. Thus, further studies are needed to understand how CLL biology contributes to CAR IEC-HS and, due to two dose-limiting toxicities (DLTs) in the CLL cohort, the dose for future CLL patients has been reduced to 1 × 10^6^ cells/kg (66). In this ongoing clinical trial, Shah et al. also examined the effect of using interleukin-7 (IL-7) and interleukin-15 (IL-15), instead of IL-2 as used in their prior study, and different manufacturing durations on the safety and efficacy of LV20.19 CAR T-cell. CAR-T cells expanded with IL-7 and IL-15 were found to be safe and effective for r/r B-cell NHL, showing a high ORR with low rates of grade 3 or higher CRS or immune effector cell-associated neurotoxic syndrome (ICANS). Early results demonstrated immunophenotypic differences and improved responses in patients treated with a shorter, 8-day manufacturing process (67). The study also reported that tandem LV20.19 CAR-T cells, expanded with IL-7 and IL-15 and manufactured onsite, were safe and effective for r/r MCL, achieving a 100% ORR at day 90, with only one relapse to date and a median follow-up of nearly two years. Thus, dual targeting of CD19 and CD20 with CAR-T cells may improve outcomes in patients with r/r MCL (68).

Another TanCAR targeting CD20 and CD19 (pLTG1497) was developed, and preclinical evaluations demonstrated improved anti-lymphoma activity. This led to the initiation of a first-in-human, phase 1 clinical study of autologous pLTG1497-transduced CAR-T cells (MB-CART2019.1, also known as Zamtocabtagene autoleucel) in patients with r/r B-NHL (NCT03870945). The goal of this phase 1 prospective multicenter trial was to determine the maximum tolerated dose (MTD) of MB-CART2019.1 in 12 adult patients with CD20- and CD19-positive r/r B-NHL, based on DLTs. No DLTs, nor CRS or neurotoxicity grade 3 or higher were observed, demonstrating excellent feasibility and safety in this cohort of elderly patients with r/r B-NHL. Sustained expansion of TanCAR-T cells was accompanied by efficacy: all patients (6/6) treated at dose level 2 (2.5 x 10^6^ cells/kg) responded, and all five patients with completed remission (5/5) remain in ongoing remission as of this report. Based on this favorable risk-benefit ratio, MB-CART2019.1 is now being evaluated at a dose of 2.5 x 10^6^ cells/kg in clinical trials for patients with relapsed aggressive B-NHL (69).

These promising results of zamtocabtagene autoleucel support the rationale for the ongoing randomized phase 2 trials, DALY 2-EU (NCT04844866) and DALY 2-USA (NCT04792489). DALY 2-EU, is a pivotal randomized, multicenter, open-label study and two arms, designed to evaluate the efficacy and safety of MB-CART2019.1 compared to standard of care (SoC) therapy in participants with r/r DLBCL who are not eligible for high-dose chemotherapy (HDC) and ASCT (70). DALY 2-USA, currently enrolling patients, investigates MB-CART2019.1 in adults with r/r DLBCL following at least two prior lines of therapy (71).

Tong et al. designed tandem CD19/CD20 CAR-T cells (TanCAR7-T cells) that form superior and stable immunological synapse structures, potentially contributing to their robust antitumor activity. In an open-label, single-arm phase 1/2a trial (NCT03097770), they enrolled 33 patients with r/r NHL, with 28 patients receiving an infusion following conditioning chemotherapy. CRS occurred in 14 patients (50%), with 36% experiencing grade 1 or 2, and 14% experiencing grade 3. One patient died from a treatment-associated severe pulmonary infection. The ORR was 79%, with a CR rate of 71%, and a PFS rate of 64% at 12 months. Notably, TanCAR7-T cells elicited a potent and durable antitumor response without causing grade 3 or higher CAR-T cell-related encephalopathy syndrome (CRES) in patients with r/r NHL (13). To further confirm these promising results and explore key covariates affecting response rates, relapse rates, and safety of TanCAR7-T cell therapy, the first 28 patients in the interim report were followed up for an extended period, and an additional cohort was recruited. These two cohorts were combined, with updated findings presented. In this trial, 87 patients with r/r NHL, including 58 with aggressive DLBCL and 24 with high tumor burden, received an infusion at doses of 0.5–8 × 10^6^ TanCAR7-T cells/kg after conditioning chemotherapy. The best ORR was 78%, with consistent response rates across prognostic subgroups. The median follow-up was 27.7 months and the median PFS was 27.6 months. CRS occurred in 61 patients (70%), with 60% of cases being grade 1 or 2, and 10% grade 3 or greater. Grade 3 CRES observed in 2 patients (2%). Three patients died due to treatment-associated severe pulmonary infections or CRS-related pulmonary injury within 1-3 months post-infusion. Long-term remissions were achieved with TanCAR7-T cells in r/r NHL, demonstrating a durable antitumor response in a heavily pretreated population, with a manageable safety profile that included CRS but few cases of CRES (72).

In another study, the efficacy and safety of autologous tandem CD19/20 CAR-T cells were investigated in eleven adult patients with r/r B cell NHL in an open-label, single-arm trial (NCT04723914). Most patients achieved a complete response. Post-infusion, the TCR repertoire diversity of CAR-T cells decreased, with expanded TCR clones *in vivo* primarily derived from those in the infusion product and showing increased expression of genes linked to immune-related signaling pathways. *In vivo* CAR-T cells kinetics were associated with upregulated genes related to immune response and cytotoxicity. In summary, this phase 1/2 trial demonstrates the safety and strong clinical efficacy of autologous CAR-T therapy targeting CD19 and CD20 in r/r NHL, underscoring the potential of dual-antigen targeting in cell-based immunotherapy. However, due to the small sample size, a larger cohort is needed to further validate the long-term efficacy and safety of this approach (14).

#### 4.2.2 TanCARs targeting CD19 and CD22

While CAR-T cell therapies targeting either CD19 or CD22 individually have shown strong anti-lymphoma effects, relapses due to antigen escape remain common. CAR-T cells targeting both CD19 and CD22 may help overcome this limitation (76).

A phase 1 clinical trial (NCT03185494) assessed the safety and feasibility of autologous CD19/CD22 CAR-T cell therapy in adult patients with r/r B-ALL. The study demonstrated that bispecific CD19/CD22 CAR-T cells could elicit a robust cytolytic response against target cells. All six enrolled patients achieved minimal residual disease-negative CR, with autologous CD19/CD22 CAR-T cells proliferating *in vivo* and being detectable in blood, bone marrow, and cerebrospinal fluid. No neurotoxicity was observed in any of the patients. However, one patient relapsed approximately five months post-treatment with blast cells lacking CD19 expression and showing reduced CD22 density. In summary, autologous CD19/CD22 CAR-T cell therapy is feasible, safe, and mediates potent anti-leukemic activity in patients with r/r B-ALL. Nevertheless, the emergence of target antigen loss and downregulation highlights the critical need to anticipate antigen escape. The clinical responses in this initial cohort have prompted expansion of the phase 1 study to evaluate the success rate, response durability, and toxicity events in a larger patient population. These results suggest that bispecific CD19/CD22 CAR-T cells help prevent antigen escape without increasing toxicity (73).

Spiegel *at al.* tested a bispecific CAR targeting CD19 and CD22 in a phase 1 clinical trial (NCT03233854) involving adults with r/r B-ALL and DLBCL. The primary endpoints were manufacturing feasibility and safety, with efficacy as a secondary endpoint. These primary endpoints were achieved: 97% of products met the protocol-specified dose, and no DLTs occurred during dose escalation. In B-ALL (n = 17), 100% of patients responded, with 88% achieving minimal residual disease-negative complete remission. In DLBCL (n = 21), 62% of patients responded, with 29% achieving complete remission. Relapses were CD19^-/lo^ in 50% (5/10) of patients with B-ALL and 29% (4/14) of patients with DLBCL but were not associated with CD22^-/lo^ disease. CD19/22 CAR products demonstrated reduced cytokine production when stimulated with CD22 compared to CD19. These results further implicate antigen loss as a major cause of CAR-T cell resistance, highlight the challenge of engineering multi-specific CAR-T cells with equivalent potency across targets, and identify cytokine production as an important quality indicator for CAR-T cell potency (74).

Zhang et al. conducted a prospective, single-arm study of bispecific CD19/22 CAR-T cells in 32 patients with r/r B-NHL (NCT03196830) to evaluate safety, efficacy, and the kinetic profiles of the CAR-T cells. The ORR was 79.3%, with a CR rate of 34.5%. The PFS and OS rates at 12 months were 40% and 63.3%, respectively. Among patients who achieved CR at 3 months, PFS and OS rates at 12 months were 66.7% and 100%, respectively. Severe CRS of grade 3 or higher occurred in nine patients (28.1%) and grade 3 or higher neurologic events occurred in four patients (12.5%). One patient died due to irreversible severe CRS-associated acute kidney injury. Responders exhibited higher maximum concentration and prolonged long-term persistence of CAR-T cells in peripheral blood. Additionally, CAR-T cell expansion within the first 28 days was associated with CRS, a key adverse event. This study highlights the safety and potential clinical efficacy of bispecific CD19/22 CAR-T cells in patients with r/r B-NHL and underscores the importance of measuring peripheral blood kinetic parameters to predict efficacy and safety in clinical applications of CAR-T cell therapy (75).

Wang et al. compared the characteristics and clinical outcomes of CD19 single-target (ChiCTR-ORN-16008948, *n* = 35) and CD19/CD22 bispecific-targeted (ChiCTR1800015575, *n*  =  15) CAR-T cells in a retrospective study of 50 patients with r/r ALL. They found that CD19/CD22 dual-target CAR-T cells had slightly lower CRS toxicity, potentially making them more suitable for elderly patients, those with severe disease, or patients with high tumor burdens. However, CD19/CD22 dual-target CAR-T cells showed a comparable CR rate to CD19 CAR-T cells and did not reduce the recurrence rate in r/r ALL (77). This CAR-T therapy was also evaluated in the same trial for r/r aggressive B cell lymphoma. They found that dual-targeted CAR-T therapy was effective, with 14 (87.5%) patients achieving an objective response and 10 (62.5%) achieving CR among the enrolled patients. The 2-year OS and PFS rates were 77.3% and 40.2%, respectively. Bispecific CAR-T therapy was safe, with only one patient experiencing severe CRS and no patients developing ICANS. These results suggest that CD19/CD22 CAR-T cells may offer a safe and potent cell-based immunotherapy for anti-lymphoma treatment (76).

Another study conducted a phase 2 clinical trial to evaluate the efficacy and toxicity of CD19/CD22 dual-targeted CAR-T therapy in patients with r/r NHL (NCT03196830). Among the enrolled patients, 33 with r/r DLBCL patients who had received DFC (decitabine, fludarabine, and cyclophosphamide) lymphodepletion chemotherapy and tandem CD19/CD22 CAR-T cells were evaluated for efficacy and toxicity. With a median follow-up of 10.9 months, the best ORR and complete remission rates were 90.9% and 63.6%, respectively. The median PFS was 10.2 months and OS was not reached. The 2-year OS and PFS rates were 54.3% and 47.2%, respectively. No grade 4 CRS was observed, grade 3 CRS occurring in 7 patients and 3 patients experienced mild ICANS. All toxicities were transient and reversible, with no CAR-T-related mortality. This study suggests that CD19/CD22 dual-targeted CAR-T therapy with a decitabine-containing lymphodepletion regimen may be a safe and effective approach for r/r DLBCL patients (78).

### 4.3 Solid tumors

#### 4.3.1 TanCARs targeting CLDN18.2 and NKG2D ligands

KD-496, a bispecific CAR-T cell therapy that simultaneously recognizes NKG2D ligands (NKG2DL) and CLDN18.2, has demonstrated superior antitumor efficacy and safety both *in vitro* and *in vivo* studies compared to monospecific CAR-T cells. KD-496 CAR-T cells have shown potent responses against gastric cancer, exhibiting more efficient tumor elimination than single-target CAR-T cells in a PDX model, with a favorable safety profile. A phase 1, single-arm, single-center, open-label study (NCT05583201) is currently underway to evaluate the safety and efficacy of KD-496 CAR-T cell infusion in patients with advanced NKG2DL/CLDN18.2-positve solid tumors (79).

## 5 Advantages and challenges for TanCAR-T cell therapy

Despite the compelling results, most patients treated with CAR-T cells ultimately experience disease progression. This can occur due to an initial lack of response, known as primary resistance, or as a result of relapse following an initial, transient response, referred to as secondary resistance. Several mechanisms of resistance to CAR-T cell immunotherapy have been identified, including CAR-T cell dysfunction, tumor-intrinsic mechanisms and immunosuppressive TME. Understanding these mechanisms is essential to developing more effective strategies that can provide durable responses in patients. Notably, the features of primary and secondary resistance after CAR-T cell therapy vary depending on the specific product and disease (80). The complex and immunosuppressive nature of the TME, tumor antigen heterogeneity, challenges in cell trafficking, CAR-T cell exhaustion, and reduced cytotoxicity at the tumor site are significant factors that limit the effectiveness of CAR-T cell therapy. These challenges underscore the need for continued advancements to enhance the performance and applicability of this treatment (81).

Advancements in technology have made bispecific CAR-T cell therapy more accessible. TanCARs offer numerous therapeutic applications, showing similar efficiency to conventional single antigen-specific CARs while being less toxic and highly effective in areas with a high disease burden (3). Key advantages include reduced manufacturing costs and greater homogeneity of cell products, which can help prevent relapses causes by target antigen loss, tumor heterogeneity, and on-target, off-tumor toxicities (25, 82).

A challenge with the simultaneous infusion of CAR-T cells is the preferential expansion of certain CAR-T cells populations targeting specific antigens, which can limit the expansion of others and impair overall efficacy. Co-transduction often results in a heterogeneous mixture of cells expressing either single CARs or dual-transduced CARs, making it difficult to achieve uniform expression levels for both CARs. Although achieving high transduction efficiencies for bicistronic CARs and TanCARs is more challenging than for monospecific CARs due to their larger construct size, this approach ensures comparable expression levels for both CARs across all transduced cells (83). When two types of CAR-T cells are co-cultured *in vivo*, they may grow disproportionately due to competition between cell populations. Connecting the two scFvs in tandem within a single CAR can prevent this growth competition. Moreover, bispecific CARs reduce the cost of producing multiple GMP-grade vectors and separately transduced T-cell lines (29, 42). A significant concern with multi-target CAR-T therapy is the high production cost. Approaches like co-administration and co-transduction involve additional viral transductions and manufacturing steps, which, when commercialized, can substantially increase the cost of therapies that are already extremely expensive (27). Therefore, compared to traditional mixed-expression CAR-T cells infusions, bispecific CAR-T cells can significantly reduce treatment costs and improve production efficiency.

Antigen escape is one of the major limitations of CAR-T cell therapy, and targeting multiple antigens with TanCARs is a potential strategy to overcome this issue (11). Antigen escape occurs when tumor cells evolve to express reduced levels of the target antigen, preventing CAR-T cells from effectively recognizing and binding to the intended target. This phenomenon can occur during or after CAR-T cell infusion and poses a significant clinical challenge. Consistent and sufficient expression of the target antigen in cancer cells is critical for optimal CAR-T cell therapy outcomes, as it affects binding affinity, activation, and cytotoxicity. When antigen loss occurs, CAR-T cells may lose their ability to effectively recognize and target tumor cells (4). Antigen escape has been identified as a mechanism of disease relapse after CAR-T cell therapy in 20-28% of patients with B-cell lymphoma, 16-68% of patients with B-ALL, and at lower rates in those with MM (84). Solid tumors have also shown evidence of antigen loss or tumor escape (85). Furthermore, certain antigens are more susceptible to loss than others. For instance, while BCMA loss in MM after CAR-T treatment is rare or only suspected, target antigen loss in solid tumors has been documented (82). Studies suggest that dual-antigen targeting, compared to single-antigen targeting, may produce synergistic responses in solid tumors, enhancing response rates and reducing the risk of antigen escape (73). Several preclinical and clinical studies have demonstrated the potential of dual-targeted CAR-T cells in addressing antigen loss. These studies have shown promising results, including improved response rates, prolonged remission, and reduced relapse rates compared with single-targeted CAR-T cell therapies (4).

However, current results are limited to small trials without comparator arms. Antigen loss still occurs in a small subset of patients, indicating that even multispecific targeting may not fully prevent target antigen loss. The role of multi-target CAR-T cell therapies in the era of immunotherapy, including bispecific antibodies and antibody-drug conjugates such as blinatumomab, loncastuximab, and inotuzumab, requires further investigation. These drugs can modify antigen expression and contribute to diseases with heterogeneous phenotypes, potentially affecting CAR-T cell therapy outcomes. Larger studies with extended follow-up are needed to evaluate safety and long-term efficacy of multispecific CARs and their ability to prevent antigen loss (26).

Despite the advantages of TanCAR-T cell therapy, challenges remain, as it does not address other resistance mechanisms beyond target antigen loss. Evidence on the safety profile and *in vivo* activity of bispecific CAR-T cells is still limited, and optimizing these complex constructs remains an unsolved task (25). This approach may also lead to excessive CAR signaling, which could promote CAR-T cell exhaustion, limit persistence, and increase the risk of adverse events. Additional limitations include the size of the TanCAR transgene, which may exceed the cargo capacity of lentiviral vectors used for transduction, as well as potential cross-linking events involving the light and heavy chain variable domains of a given TanCAR (6).

It is challenging to bind two different antigens, as the corresponding antigen-binding fragments must be effectively engineered into T cells to produce a bispecific CAR structure in the T cell membrane. This process involves designing appropriate CAR constructs, generating viral packaging vectors, producing viruses for transduction, and establishing bispecific CAR-T cells through viral transduction. Transduction efficiency is a major issue that requires improvement (42). The design and construction of CAR-T cells in both bicistronic CAR and TanCAR strategies face obstacles, and the size of the constructs is limited by the packaging capacity of the viral vector (29). The length of co-stimulatory domains and linkers needed to create a bispecific CAR construct requires a large-sized vector, complicating viral packaging and reducing transduction efficiency. Poor transduction efficiency results in reduced expression of bispecific CAR constructs on the T cell membrane (25). For bicistronic CARs, one challenge is that expressing two CAR molecules in a viral bicistronic plasmid may require codon optimization of duplicated DNA sequences (e.g., CD3ζ) to reduce the risk of DNA recombination. TanCARs offer an alternative, fusing two scFvs with different antigen specificities in the extracellular domain and connecting to a single intracellular signaling module. TanCARs have the advantage of a smaller transgene size (approximately 40% shorter than bicistronic CARs), which is particularly beneficial when incorporating additional transgenes, such a safety switches or cytokines, into the CAR plasmid. This is critical, as previous studies have shown that construct size impacts viral vector packaging and transduction efficiency (44).

Constructing a TanCAR requires additional considerations, such as potential cross-pairing between the VL and VH sequences of different scFvs and the length of the extracellular spacer. Besides transduction efficiency, the spatial arrangement of the two scFvs also influences bispecific CAR-T cell activity, making it necessary to explore optimal CAR-T cell structure for recognizing specific targets cells. In constructing CD19/CD20 TanCARs, Zah et al. proposed arranging the CAR construct as scFv1 (VL-VH)-scFv2 (VH-VL) to minimize potential cross-pairing in the VL and VH domains between the two scFvs. Regarding the extracellular spacer length, shorter spacers demonstrated better activity in anti-CD19 CARs, whereas longer spacers were most effective for CD20. Therefore, the spacer length and targeted epitope position must be adjusted based on the specific antigen characteristics (29). Other studies on CD19/CD20 and CD19/CD22 bispecific CAR-T cells have shown that a shorter distance between the scFv and the target on the cell membrane can lead to higher CAR-T cell activity, supporting the development of a bivalent vector encoding a short linker to connect the two scFvs in bispecific CARs (25). TanCAR design requires fine-tuning of spacer length and linker sequence between scFvs, as well as careful orientation to optimize antigen recognition and T cell activation. Computational modeling can assist in the design of these TanCARs, improving their structural predictions and facilitating their development (74, 86).

The findings and recent research on TanCARs could have significant implications for future research directions, including the development of next-generation CAR-T cells and combinatorial therapies. Multiple biological limitations of currently approved CAR-T cells can potentially be addressed through innovative engineering strategies and combination therapies. Combining CAR-T cells with treatments that sustain target antigen expression on the tumor surface is a promising strategy to prevent antigen downregulation. Additionally, new-generation CAR-T cells are being engineered to secrete molecules such as cytokines to enhance antitumor activity (80). To the best of our knowledge, advances in CAR-T cell combination therapies with other therapeutic approaches have opened promising horizons for more effective cancer treatments, particularly for solid tumors. Various studies have supported this idea, demonstrating that combination therapies significantly improve the effectiveness of CAR-T cell therapy while reducing its side effects (81). Furthermore, novel CAR designs, coupled with advancements in gene transfer and editing technology, hold the potential to increase access to engineered cell therapies and improve their potency in solid tumors. Remarkable progress in molecular biology, immunology, gene editing, synthetic biology, and computational analysis provide powerful new tools for CAR T cell development. These advances are critical to achieving a greater therapeutic success and expanding the use of CAR-T cell therapies to a broader range of cancer types (86).

In summary, challenges in TanCAR-T cell manufacturing include the complex optimization process to selecting suitable vectors, inconsistencies in batch viral vector manufacturing, low transduction efficiency in bispecific CAR-T cells, and a high manufacturing failure rate due to the size of the bivalent and bicistronic vector. These issues need to be resolved (25).

## 6 Conclusions

Dual-targeting CAR-T cell therapy, a rapidly advancing form of tumor immunotherapy, offers new hope for patients beyond the era of monospecific CAR-T cell therapy. The clinical efficacy of TanCAR-T therapy has been validated in several trials; however, the data on multi-targeting strategies remain limited, and many challenges still need to be addressed. An optimal bispecific CAR structure has yet to be established, highlighting the need for further optimization. This process should focus on identifying appropriate targets for different indications, designing an optimal spatial arrangement of the two distinct scFvs, selecting suitable linker for the scFvs, and ensuring efficient transduction using patient-derived T cells to enhance the efficacy and persistence of TanCAR-T cells. The absence of a perfect bispecific CAR structure underscores the importance of collaboration among research groups to develop solutions that benefit the global community. Integrating clinical and preclinical data into predictive models can guide the design of an ideal vector for TanCAR therapy. Although current results are promising, additional clinical trials are underway. Ongoing collaborative research and development efforts aim to improve TanCAR-T cell therapy, making it a more accessible and effective treatment option for cancer.
